# Complete genome sequence of the *Bombyx mori* nucleopolyhedrovirus strain P6E

**DOI:** 10.1128/mra.00413-25

**Published:** 2025-07-08

**Authors:** Tsuneyuki Tatsuke

**Affiliations:** 1 Institute of Agrobiological Sciences, National Agriculture and Food Research Organization 13516, Tsukuba, Ibaraki, Japan; DOE Joint Genome Institute, Berkeley, California, USA

**Keywords:** baculovirus, insect viruses

## Abstract

This study reports the complete genome sequence of *Bombyx mori* nucleopolyhedrovirus strain P6E, used as a protein expression vector. The genome is 126,727 bp long, with a G + C content of 40.2% and 141 open reading frames. This strain has a unique combination of *bro*-paralog genes compared with other Japanese strains.

## ANNOUNCEMENT

*Bombyx mori* nucleopolyhedrovirus (BmNPV), a member of the family *Baculoviridae* and genus *Alphabaculovirus*, possesses an approximately 130 kb circular double-stranded DNA genome that encodes approximately 140 proteins. This virus is an important pathogen that specifically infects domesticated and wild mulberry silkworms, resulting in considerable economic losses in sericulture. Conversely, a protein expression system utilizing silkworms and BmNPV was initially reported by Maeda et al. (1), and has been employed to produce valuable proteins for research and industrial applications.

This study reports the complete genome sequence of the BmNPV strain P6E, which was modified and utilized by Kobayashi et al. (2) at the National Institute of Sericultural and Entomological Science (NISES) as a protein expression vector for the early development of a silkworm baculovirus expression system (3, 4). At that time, sequencing the entire genome of the virus required significant effort, which had not yet been fully completed.

The virus, isolated from *B. mori* larvae, maintained at NISES and plaque-purified (2), was propagated in BmN cells. Budded virus (BV) and occlusion-derived virus (ODV) were collected from the culture supernatants and infected cells, respectively. DNA was extracted from BV (untreated) and ODV (following sodium carbonate treatment) using phenol-chloroform and isopropanol precipitation (5).

A long-read library was constructed from 500 ng of viral genomic DNA extracted from BV using the Ligation Sequencing Kit (SQK-LSK109) without shearing and size selection. The library was sequenced on an Oxford Nanopore Technologies MinION Mk1B using a Flongle Flow Cell (R9.4.1) (5), producing 19.8k reads. The Nanopore read *N*_50_ is 16.26 kb. Following super-accuracy basecalling, DNA sequences excluding reads less than 20 kb were *de novo* assembled using Flye 2.9.2 (6) and polished using medaka 1.8.0. The assembly yielded a single circular contig with genome read coverage of 172. A short-read library was constructed from viral genomic DNA from the ODV using the TruSeq DNA PCR-Free (Illumina, CA, USA) according to the manufacturer’s instructions (TruSeq DNA PCR-Free Sample Preparation Guide, Part # 15036187 Rev. D) and sequenced on an Illumina NovaSeqX, outsourced to Macrogen Japan Corp. (Kyoto, Japan). Error correction of the *de novo* assembled sequence was repeated with polca (7) until the results converged using short-reads result, approximately 31.7 million reads (4.8 Gbp) of 150 bp paired-end reads. The resulting complete genome sequence was annotated by blastn using the CDS sequences of the BmNPV strain T3 (GenBank accession number L33180) as a reference (8) and manually curated. All tools were run with default parameters unless otherwise specified.

The genome of the BmNPV P6E consists of 126,727 bp with a G + C content of 40.2% and 141 open reading frames (ORFs), including ORFs for the 38 core genes of *Baculoviridae* (9, 10). The genome is 98.91% identical to that of BmNPV T3 from blast2seq result (11) and includes three *bro* genes (*bro-a*, *bro-c*, and *bro-d*) and eight-*hr* regions (*hr1*, *hr2L*, *hr2R*, *hr3*, *hr4a*, *hr4b*, *hr4c*, and *hr5*) (8). However, the combination of *bro*-paralog genes in BmNPV P6E is unique and differs from other strains found in Japan. This combination matches that of BmNPV strains C2, Zhejiang, and India, as well as BomaNPV strain S1, isolated from the Asian continent (12).

## Data Availability

The genome sequence (LC867272) and raw reads (DRR657489–DRR657490) are available in the DDBJ/EMBL/GenBank and DDBJ Sequence Read Archive, respectively.
